# Testing Differential Holistic Processing Within a Face: No Evidence of Asymmetry from the Complete Composite Task

**DOI:** 10.3389/fpsyg.2016.01506

**Published:** 2016-10-04

**Authors:** Gary C.-W. Shyi, Chao-Chih Wang

**Affiliations:** ^1^Department of Psychology and Center for Research in Cognitive Science, National Chung Cheng UniversityChia-Yi, Taiwan; ^2^Advanced Institute of Manufacturing with High-tech Innovations, National Chung Cheng UniversityChia-Yi, Taiwan

**Keywords:** face recognition, holistic processing, asymmetry, congruency effect, perceptual field hypothesis

## Abstract

The composite face task is one of the most popular research paradigms for measuring holistic processing of upright faces. The exact mechanism underlying holistic processing remains elusive and controversial, and some studies have suggested that holistic processing may not be evenly distributed, in that the top-half of a face might induce stronger holistic processing than its bottom-half counterpart. In two experiments, we further examined the possibility of asymmetric holistic processing. Prior to Experiment 1, we confirmed that perceptual discriminability was equated between top and bottom face halves; we found no differences in performance between top and bottom face halves when they were presented individually. Then, in Experiment 1, using the composite face task with the complete design to reduce response bias, we failed to obtain evidence that would support the notion of asymmetric holistic processing between top and bottom face halves. To further reduce performance variability and to remove lingering holistic effects observed in the misaligned condition in Experiment 1, we doubled the number of trials and increased misalignment between top and bottom face halves to make misalignment more salient in Experiment 2. Even with these additional manipulations, we were unable to find evidence indicative of asymmetric holistic processing. Taken together, these findings suggest that holistic processing is distributed homogenously within an upright face.

## Introduction

Face recognition is a ubiquitous ability for humans and many investigators agree that at its core is holistic processing (Tanaka and Farah, 1993; McKone, 2010). Using the composite face task, Young et al. (1987) were among the first to demonstrate holistic processing of faces, which many regard as the hallmark of face processing and which is at the core of the debate between the expertise hypothesis and domain-specificity hypothesis of face processing (Kanwisher, 2000; Gauthier and Tarr, 2002; Gauthier et al., 2010; McKone, 2010). The composite task has been used to assess failures of selective attention to irrelevant face parts, and failures in selective attention result in unwarranted processing of irrelevant parts, which in turn interferences with processing of target face parts. Participants cannot focus on the specific part (e.g., the top face half) while ignoring the irrelevant part (e.g., the bottom face half), which implies that faces are processed holistically, rather than as parts that are combined. Young et al. (1987) designed the composite task and used celebrity faces as stimuli. Participants were asked to name celebrities based on the top-half of composite faces, and the bottom face half interfered with performance more in the aligned (composite) than misaligned (non-composite) condition. In other words, it was more difficult for participants to respond to the same celebrity in the top face half in the aligned than misaligned condition. Based on these findings, Young et al. (1987) suggested that for aligned faces, participants perceive integration of the top and bottom face parts, and such integrated, holistic processing is disrupted with misaligned faces. Young et al. (1987) concluded that processing face identity requires holistic processing, not merely featural processing. It is interesting to note that they used inverted faces instead of misaligned faces in their second experiment and found comparable results, suggesting that face inversion might share the same mechanisms with (or at least be functionally equivalent to) misalignment in terms of disrupting holistic processing (Gauthier and Bukach, 2007).

Following Young et' al. (1987) initial study, Hole (1994) demonstrated that irrelevant face parts also influence simultaneous matching of unfamiliar faces. In each trial, a pair of faces was simultaneously presented and observers had to judge whether the top parts of the displayed faces were the same or different by a button press. This differs from the naming task used by Young et al. (1987). Regardless of task, findings from these earlier studies lend support to the conjecture that upright faces are processed holistically rather than via piecemeal featural processing.

Over the past two decades, researchers have found support for the notion that holistic processing plays a central role in face perception and recognition (Gauthier et al., 1998; Gauthier and Tarr, 2002; Robbins and McKone, 2003, 2007), and many are now trying to answer the question regarding the exact nature of holistic processing and its underlying mechanisms (for reviews, see Rossion, 2013; Richler and Gauthier, 2014). Currently, there are two main hypotheses, the template hypothesis (also called the holistic encoding hypothesis) and the attention strategy hypothesis (Richler and Gauthier, 2014). According to the template hypothesis, faces are encoded as a single unit to fit a template (Tanaka and Farah, 1993; Farah et al., 1998). The whole face is matched to a unified memory template rather than to parts. In other words, faces are represented as an undifferentiated whole because facial features are glued into a single unitary representation (Richler et al., 2012). Alternatively, the attention strategy hypothesis proposes that faces are processed holistically because attention to the whole becomes automatized with experience (Richler et al., 2011b, 2012). In other words, while facial features could be encoded and represented independently, holistic processing arises from a strategy of attending to all face parts simultaneously.

**FIGURE 1:**
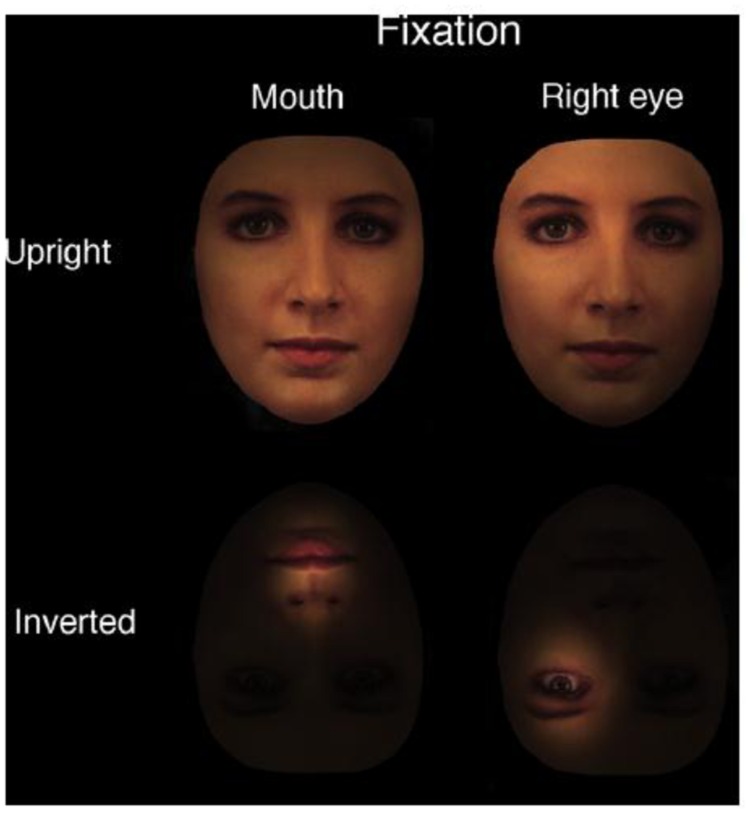
**According to the perceptual field hypothesis, an upright face is perceived as an integrated, whole face, rather than a collection of local features due to an expanded perceptual field that encompasses the entire face (upper left quadrant).** In contrast, an inverted face is perceived as a collection of local features, rather than an integrated, whole face, due to the contraction of the perceptual field (adapted from Rossion, 2009, with permission).

In addition to these two views, Rossion (2008, 2009, 2013) and van Belle et al. (2010) proposed the perceptual field hypothesis, which in their view is compatible with the holistic encoding or template hypothesis, to explain the inversion effect in face processing (Rossion, 2013). Specifically, as illustrated in **Figure 1**, the perceptual field for an upright face is expanded to cover almost the entire face, which results in an observer’s perception of a whole face, rather than a collection of facial features in isolation (Rossion, 2009; van Belle et al., 2010). When faces are inverted, however, the perceptual field is contracted to contain only specific local features, and observers perceive one local feature at a time (**Figure 1**). This hypothesis has been used to explain inversion effects in face perception. For example, using gaze-contingent displays, van Belle et al. (2010) showed that the difference between upright and inverted faces disappears when observers could only perceive a face one piece at a time through a gaze-contingent window that necessarily disrupts holistic processing.

The face template, attention strategy, and perceptual field hypotheses might not be completely incompatible with one another in terms of symmetry of holistic processing within a face. All three hypotheses emphasize integration of facial features into some sort of holistic representation during face processing, and posit that it is difficult, if not impossible, to process features independently in upright faces. However, the three hypotheses differ in terms of the origin of holistic processing. Whereas the attention strategy and perceptual field hypotheses emphasize the influence of experience from regular exposure to faces and frequent social interactions (Rossion, 2013; Richler and Gauthier, 2014), the template hypothesis postulates an internal origin for holistic processing. Specifically, the face template may be established innately, and its impact can be observed during early infancy (McKone et al., 2007; McKone, 2010).

One implication of the perceptual field hypothesis (Rossion, 2009, 2013) is that participants perceive upright faces in entirety rather than a combination of top and bottom halves, even though they are able to pay attention to the top or bottom half upon request. Therefore both the template and perceptual field hypotheses appear to assume homogeneous or unitary holistic processing within an upright face. Consequently, both hypotheses predict that comparable holistic effects should be observed regardless of whether top or bottom face halves are targets. In contrast, according to the attention strategy hypothesis, holistic processing is a failure of selective attention in the composite task (Richler et al., 2009, 2012; Richler and Gauthier, 2014), and it is unclear whether attentional weights for top and bottom parts are equal. If they are, then holistic processing should be symmetrical; if weights are not equal, holistic processing should be asymmetrical. In fact, a recent study by Chua et al. (2014) showed that attentional weights to different face parts (and hence holistic processing) can be modulated via learning to pay attention to either the top, bottom, or both face parts based on which part or parts were diagnostic for differentiating group members.

Alternatively, processing resources may not be evenly distributed within a face, leading to the prediction of asymmetrical holistic processing in the composite face task. In fact, empirical evidence reviewed by Rossion (2013) suggests that the face research field at large appears to be in favor of a top/bottom asymmetry in holistic processing. The first empirical evidence purportedly supporting asymmetric holistic processing was from Young et al. (1987), where reaction times (RT) for naming were longer for top versus bottom face halves in the composite (aligned) condition. Furthermore, the magnitude of the alignment effect (difference in RT between composite (aligned) and non-composite (misaligned) conditions) was greater for top (256 ms) than bottom (159 ms) face parts.

More generally, Rossion (2013) offered three possible explanations for asymmetry in holistic processing. First, the top part (e.g., eyes and eyebrows) are more important than the bottom part when recognizing identity. Second, the location of optimal fixation for the purpose of identifying a face is in the top part. Third, the top part includes more elements (two eyes, eyebrows, part of nose) than the bottom part (essentially a single mouth). Although these putative possibilities sound reasonable, it is important to re-evaluate the results of Young et al. (1987) more carefully before accepting the asymmetry hypothesis. Specifically, mean RTs in the non-composite (misaligned) condition were shorter for top (1041 ms) than bottom (1123 ms) parts, even though top (1297 ms), and bottom (1282 ms) parts yielded comparable RTs in the composite condition, suggesting that it was easier for participants to compare misaligned top parts than misaligned bottom parts. Therefore, the results from misaligned (or non-composite) trials fail to provide a baseline control for differential holistic processing between top and bottom parts on aligned (composite) trials.

In a more recent study, Schwartz et al. (2002) reported a reverse finding, such that holistic processing (measured in terms of an RT difference) was larger when the bottom face part was the target than when the top face part was the target. However, closer inspection reveals that their results might be driven by ceiling effects. Specifically, accuracy for top parts was 98% in the aligned condition and 99% in the misaligned condition, yielding a relatively small alignment effect (i.e., 1%). In contrast, accuracy for bottom parts was 87% in the aligned condition and 91% in the misaligned condition, resulting an alignment effect of 4%. The near-perfect performance for top parts in the misaligned condition clearly suggest that top face halves were easily discriminable compared to bottom face halves, which may confound the baseline control for inferring differential holistic processing. We think it is important to control relative discriminability between top and bottom face halves before assessing the possibility of differential holistic processing within a face.

Finally, some studies have demonstrated that the eye region is more diagnostic than the mouth region, and suggest that it is easier to detect the eye versus mouth region (Davies et al., 1977; Haig, 1985; Gosselin and Schyns, 2001). For example, Davies et al. (1977) asked participants to select which of six faces matched a target face. Participants were more likely to erroneously choose faces that had different mouths from the target versus different eyes. In other words, eyes were more salient face cues, such that participants were more likely to notice if they changed.

Taken together, these findings suggest that the conclusion that there is a top/bottom asymmetry in face processing (Rossion, 2013) may be at least partially due to uneven discriminability between the two face halves. Therefore, in the present study we examined the possibility of differential holistic processing within a face without confounding relative discriminability. Specifically, we first confirmed that the discriminability of top and bottom face parts was equal. Then, we used the *complete* composite task (Gauthier and Bukach, 2007) to test whether there is differential holistic processing within a face.

**FIGURE 2:**
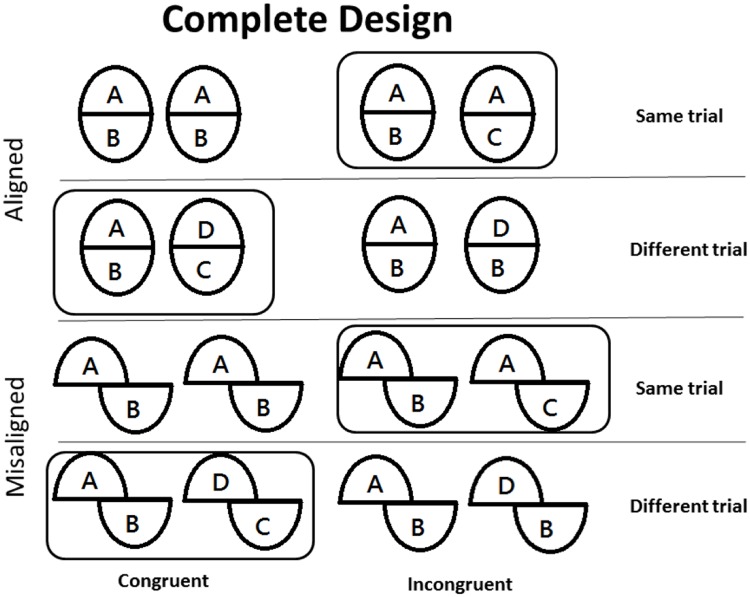
**Illustration of the complete design that includes partial design trials.** Capital letters denote face part identity, where the same letters indicate identical face halves, and different letters indicate different face halves. Many researchers have used the partial design, denoted by black surrounding frames, to demonstrate holistic processing, but in the partial design the irrelevant (bottom) parts were always different. Moreover, only performance on “same” trials were analyzed. In contrast, in the complete design, congruency effects, which include both “same” and “different trials,” are computed as a measure for holistic processing.

Both Young et al. (1987) and Hole (1994) only calculated the difference in performance between aligned and misaligned conditions (alignment effect) for trials where top halves were the same, while completely ignoring data from different trials (Robbins and McKone, 2007; Rossion, 2013). Gauthier and Bukach (2007) proposed what they called the *complete* design to replace this traditional composite task, also called the *partial* design, for two reasons. First, although some researchers have suggested that only data from same trials in the partial design should be analyzed (Robbins and McKone, 2007; Rossion, 2013) (**Figure 2**), Gauthier and Bukach (2007) and Richler and Gauthier (2014) argued that data from both same and different trials should be analyzed, because both are relevant for explaining the composite illusion. When different trials are ignored in the partial design, it is impossible to determine whether irrelevant parts facilitate or interfere with performance when relevant parts are different.

The second and perhaps more critical reason is that the partial design is susceptible to response biases (Richler and Gauthier, 2014) because participants tend to respond “same” more often in the upright face condition than in the inverted face condition (Farah et al., 1998; Wenger and Ingvalson, 2003) and in the aligned condition than the misaligned condition (Gauthier and Bukach, 2007). Moreover, participants are more likely to respond “same” on trials where relevant and irrelevant parts are both “same” or both “different” (congruent trials) than trials where one part is the same and the other is different (incongruent condition), but in the partial design correct response and congruency are confounded (all “same” trials are incongruent and all “different” trials are congruent).

To rule out these potential problems, Gauthier and Bukach (2007) proposed that holistic processing should be assessed and measured in terms of a *congruency* effect (i.e., difference in performance between the congruent and incongruent conditions) using sensitivity (*d’*) as the dependent variable (Green and Swets, 1966). Sensitivity is better on congruent than incongruent trials in the aligned condition, and the magnitude of this congruency effect is reduced in the misaligned condition (Richler and Gauthier, 2014, for a meta-analysis). We think it is appropriate to use the complete design of the composite task.

In the present study, we calculated congruency effects as an index of holistic processing, and expect to find an interaction between congruency and alignment, where the congruency effect will be larger for aligned than misaligned faces (Richler et al., 2011a; Wong et al., 2012; Richler and Gauthier, 2014). Moreover, to avoid potential confounds, we verified that perceptual discriminability was equivalent between the top and bottom face halves used in Experiment 1.

## Experiment 1

The goal of Experiment 1 was to test whether the magnitude of holistic processing would differ when the top versus bottom face half was the target in the complete design of the composite task (Gauthier and Bukach, 2007; Wong et al., 2012). Prior to the composite task, we verified that top and bottom face halves were equally discriminable.

### Materials and Methods

#### Participants

Sixteen college students (6 male, 10 female) from the National Chung Cheng University participated in Experiment 1 for NT$ 100. Mean age was 21.5 years (*SD* = 2.56, range = 18–25 years). All participants had normal or corrected to normal vision. Participants were recruited in accordance with approval of the Research Ethics Committee of National Chung Cheng University, Chia-Yi, Taiwan.

#### Design

We adopted the complete design and computed a measure of sensitivity (*d’*) for each participant as the dependent variable. “Same” responses on “same” trials were defined as *hits*, and “same” responses on “different” trials were defined as *false alarms*. In each trial, two composite faces were shown simultaneously. The top or bottom part was designated as the target for each block. For aligned composites, the top and bottom face halves were modified slightly whenever necessary to create smooth alignment between the two halves. For misaligned composites, top and bottom face halves were moved horizontally. The same face stimuli were used for aligned and misaligned conditions regardless of whether the target was the top or bottom face half.

#### Stimuli

For face stimuli, we first created 32 different Asian face images with equal number of male and female faces using *FaceGen 3.1* (Singular Inversions, Canada). Half (eight male and eight female) were designated as the target set, and the remaining half were designated as the irrelevant set. To ensure that top and bottom face halves were equally discriminable, we tested another group of 14 college students (six female, eight male) from the National Chung Cheng University in a task where face halves (top or bottom) were presented alone. A pair of face halves were presented in each trial, and participants were asked to judge whether or not the two halves were identical. Each participant completed eight practice trials and 256 formal trials, which took about 20 min. Mean performance for top face halves (*M* = 2.07) was almost identical to mean performance for bottom face halves (*M* = 2.17), *t*(13) = 0.675, *p* > 0.05, suggesting that the face halves were equally discriminable. These face halves were then used to construct face composites.

Top halves from the relevant set were randomly paired with bottom halves from the irrelevant set to create face composites in accordance with the complete design illustrated in **Figure 2**. Specifically, there were 16 faces for each of the four face composites (A/B, A/C, D/C, and D/B) in **Figure 2**.

Each face image was shown in grayscale on a black square background with 100 pixels on each side. When presented on the display screen, each face was about 4.01 cm in width and 4.80 cm in height, subtending a visual angle of about 5.10° × 6.11° at a viewing distance of approximately 45 cm. An overextended white line was overlaid horizontally in the middle of each face to clearly demark the top and bottom halves. The line was of 8.18 cm in length and 0.14 cm in height, subtending 10.39° × 0.18° of visual angle. The white line did not disrupt the perceptual integrity of the face, but was necessary to clearly distinguish the top and bottom halves (Rossion, 2013). The top and bottom halves of faces were separated by about 2° of visual arc (25 pixels) in the misaligned condition.

#### Procedure

**FIGURE 3:**
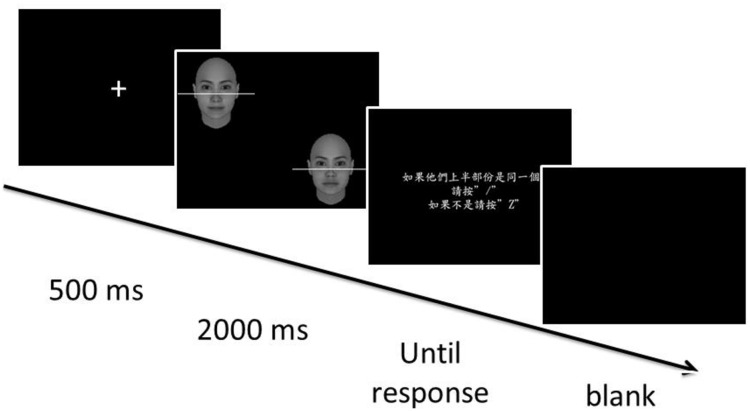
**Illustration of events in a single trial for Experiment 1**.

In each trial (**Figure 3**), a fixation cross was shown for 500 ms, followed by the presentation of a pair of composite faces for 2000 ms. Participants were asked to judge whether the top halves of the faces were identical while ignoring the bottom halves, or vice versa. One face was located in the upper left quadrant and the other face was located in the lower right quadrant to discourage feature-by-feature comparison. The center of the face in the left quadrant was about 4.69 cm (6° in visual arc) below the top edge of the monitor, and about 13.31 cm (visual angel 16.83°) from the left edge of the monitor. The center of the face in the lower-right quadrant was roughly the same distances from the bottom and right edge of the monitor. The two faces were separated by a center-to-center distance of about 14.12 cm (17.83° in visual arc). The top half was the target in one block, and the bottom half was the target in another block. The order of the two blocks was counterbalanced across participants. It took about 40 min for participants to complete 24 practice trials and 256 experimental trials. Note the same set of 256 composite images was used in both blocks.

### Results and Discussion

**FIGURE 4:**
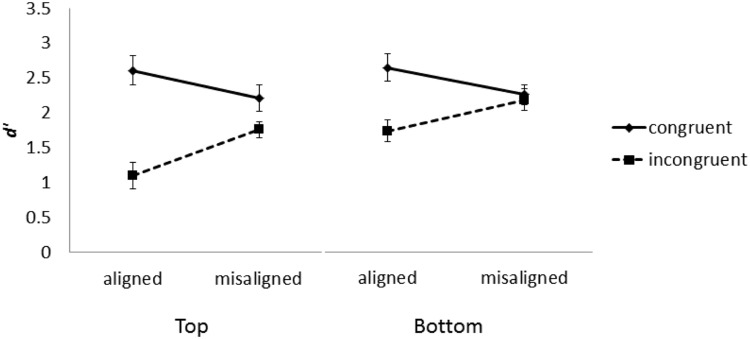
**Performance in the complete composite task for top and bottom halves in Experiment 1.** Error bars indicates ±1 standard error of mean.

Mean *d*’ was computed in each condition and submitted to a three-way repeated-measure ANOVA with part (top vs. bottom), congruency (congruent vs. incongruent), and alignment (aligned vs. misaligned) as within-participants variables. As illustrated in **Figure 4**, the main effects of congruency and part were both significant, *F*(1,15) = 5.01, *MSE* = 2.60, *p* < 0.05, and *F*(1,15) = 44.27, *MSE* = 17.41, *p* < 0.001, respectively. The performance in the bottom part condition (*M* = 2.21) was better than that in the top part condition (*M* = 1.92) and the performance on congruent trails (*M* = 2.43) was better than that on incongruent trials (*M* = 1.70). The two-way interactions between part and congruency, *F*(1,15) = 6.57, *MSE* = 1.92, *p* < 0.05, and between alignment and congruency, *F*(1,15) = 30.56, *MSE* = 7.13, *p* < 0.001, were also significant. The difference between the congruent trials and incongruent trials in the top part condition (*M* = 0.98) was greater than that in the bottom part condition (*M* = 0.49). The difference between congruent trials and incongruent trials in the aligned condition (*M* = 1.21) was greater than that in the misaligned condition (*M* = 0.27). However, the three-way interaction between part, alignment, and congruency was not significant, *F* < 1.

**FIGURE 5:**
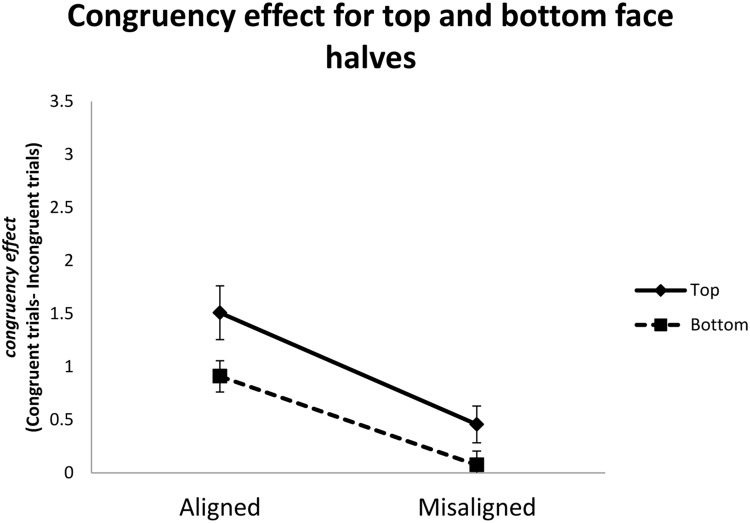
**Congruency effects, defined as differences in *d*’ between congruent and incongruent trials, as a function of face part (top vs. bottom) and alignment (aligned vs. misaligned) in Experiment 1.** Error bar indicated ±1 standard error of mean.

The two-way interaction between alignment and congruency is consistent with many previous studies (Richler and Gauthier, 2014), indicating that the irrelevant parts were less likely to affect relevant parts in the misaligned (*M* = 0.27) than aligned condition (*M* = 1.21) because spatial misalignment disrupts holistic processing. To further examine the possibility of differential holistic processing, we submitted the difference in *d*’ between congruent and incongruent trials (congruency effect) to a two-way repeated-measure ANOVA with part and alignment as independent variables. As illustrated in **Figure 5**, both the main effects of part and alignment were significant, *F*(1,15) = 6.57, *MSE* = 3.85, *p* < 0.022, and *F*(1,15) = 30.56, *MSE* = 14.27, *p* < 0.001. The congruency effect for the top part condition (*M* = 0.49) was greater than those for the bottom part condition (*M* = 0.23). The congruency effect in the aligned condition (*M* = 0.60) was greater than that in the misaligned condition (*M* = 0.13). However, the interaction between part and alignment was not significant, *F* < 1. Therefore, we found no evidence for differential holistic processing between the top and bottom parts.

Although the three-way interaction between part, alignment, and congruency, which would be indicative of asymmetry in holistic processing between top and bottom face halves, was not significant, it is worth noting that the two-way interaction between part and congruency was significant. Follow-up analyses showed that there was a difference between the top and bottom parts on incongruent, but not congruent trials *F*(1,15) = 10.54, *MSE* = 2.25, *p* < 0.01, and *F* < 1, respectively. These results suggest that the top and bottom parts might not be equally discriminable.

However, these findings do not necessarily mean that we failed to control perceptual discriminability between the top and bottom parts. Rather, a more plausible explanation may have to do with the fact that face halves with equivalent discriminability when presented in isolation were positioned together to create whole faces. Instructions to respond to the target part while ignoring the irrelevant part may not completely prevent perceptual input from the latter while participants presumably focused processing on the former. As Rossion (2009) and van Belle et al. (2010) predict, the perceptual field likely encompasses the entire face when it is presented upright (compared to when it is inverted). Moreover, although the face features included in the perceptual field may be identical regardless of which part is the target when the two parts are aligned, this may not be the case when the two parts are separated in the misaligned condition.

**FIGURE 6:**
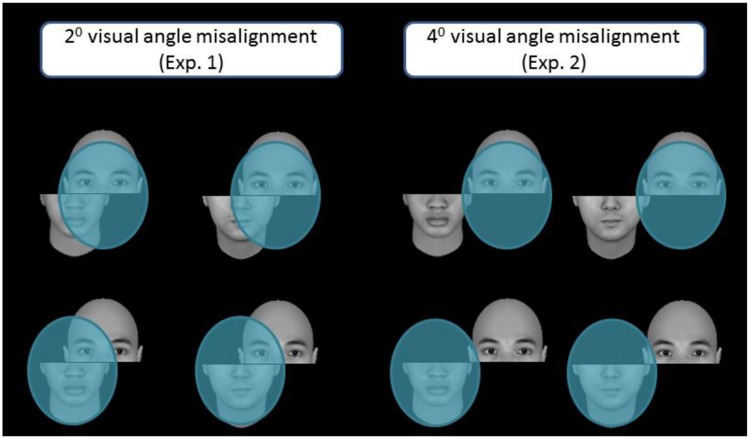
**An illustration of the perceptual field (blue ovals) and its distribution in the misaligned condition when either the top part (top row) or bottom part (bottom row) was the target.** Note the blue ovals presumably depict the extent of the perceptual field for upright faces. When participants focus on the top part, the perceptual field contains more facial features than when they focus on the bottom part. Note also the difference in the content of the perceptual field when the top and bottom parts were moderately separated, as in Experiment 1 (left half of the figure), as opposed to when they were completely separated, as in Experiment 2 (right half of the figure).

As illustrated in left half of **Figure 6**, when the top of a face is the target, the perceptual field may contain more facial details than when the bottom part is the target. This difference may be more disruptive to performance on incongruent trials, where top and bottom parts elicit contrasting responses, than congruent trials, where the two parts elicit identical responses. These differences may have contributed to the interaction between part and congruency in Experiment 1. In fact, inspection of **Figure 4** suggests that both aligned and misaligned conditions yielded comparable performance for top and bottom parts in congruent trials, and performance differed between top and bottom parts in based on alignment in incongruent trials.

## Experiment 2

As a better control for the potential confound discussed above, we further displaced top and bottom parts so that they were completely separated in the misaligned condition (see right side of **Figure 5**), which may additionally serve to eliminate lingering holistic effects observed in that condition of Experiment 1 (**Figure 4**). It is worth noting that in Experiment 1, variability of the congruency effect for the aligned condition when top half was the target was relatively large compared to the other conditions (**Figure 5**). To reduce performance variability, we doubled the number of trials in Experiment 2.

### Materials and Methods

#### Participants

Nineteen college students (9 male, 10 female) from the National Chung Cheng University in Chiayi County, Taiwan, participated in Experiment 2. All participants had normal or corrected to normal vision, and received NTD$120 for their participation.

#### Stimuli

Stimuli were the same as Experiment 1, except we increased the separation between top and bottom face halves in the misaligned condition. The top part was displaced to the right by about 4° visual angle in Experiment 2, which is double the displacement used in Experiment 1.

#### Procedure

The procedure was identical to Experiment 1. Each participant completed eight practice trials and 512 formal trials, which took about 50 min.

### Results and Discussion

**FIGURE 7:**
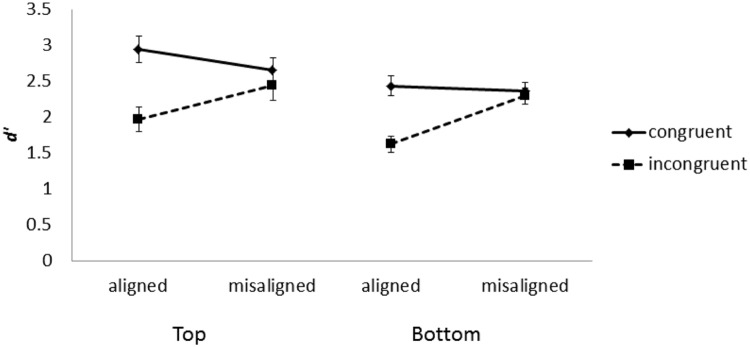
**Mean *d*’ as a function face part, alignment, and congruency in Experiment 2.** Error bars indicate ±1 standard error of mean.

As illustrated in **Figure 7**, mean *d*’ was computed in each condition and submitted to a three-way repeated-measure ANOVA with part, congruency, and alignment as within-participant variables. The main effects of part, alignment, and congruency were significant, *F*(1,18) = 4.56, *MSE* = 3.94, *p* < 0.05, *F*(1,18) = 10.08, *MSE* = 1.51*, p* < 0.001, and *F*(1,18) = 67.52, *MSE* = 10.22, *p* < 0.001, respectively. The performance in the top part condition (*M* = 2.51) was better than that in the bottom part condition (*M* = 2.18). The performance in the misaligned condition (*M* = 2.45) was better than that in the aligned condition (*M* = 2.24). The performance on congruent trials (*M* = 2.61) was better than that on incongruent trials (*M* = 2.09). The two-way interaction between alignment and congruency also was significant, *F*(1,18) = 22.38, *MSE* = 5.33, *p* < 0.001. The difference between congruent trials and incongruent trials in the aligned condition (*M* = 0.45) was greater than that in the misaligned condition (*M* = 0.07). However, the three-way interaction was not significant, *F* < 1. As indicated in **Figure 7**, the interaction between congruency and alignment was very similar regardless of whether the top or bottom half was the target. Contrary to Experiment 1, the interaction between part and congruency was not significant, *F*(1,18) = 1.28, *MSE* = 0.22, *p* > 0.05.

**FIGURE 8:**
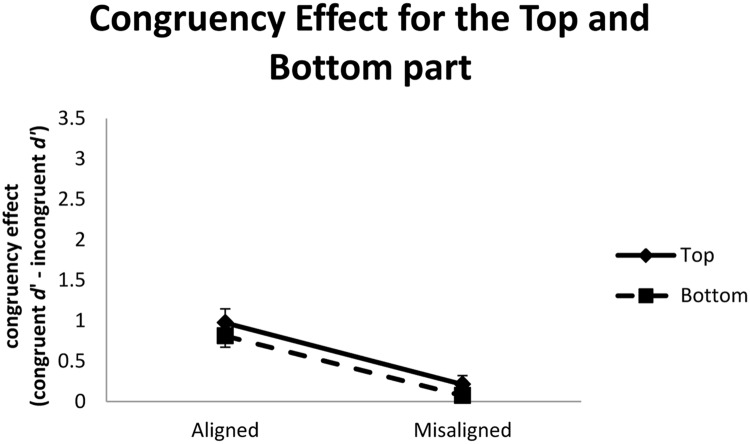
**Congruency effects, defined as the difference in *d*’ between congruent and incongruent trials, as a function of face part (top vs. bottom) and alignment (aligned vs. misaligned) in Experiment 2.** Error bars indicate ±1 standard error of mean.

This observation was further confirmed when we used magnitude of congruency effect (i.e., difference in *d*’ between congruent and incongruent trials) as the dependent variable and performed a two-way repeated-measure ANOVA with part and alignment as independent variables. As shown in **Figure 8**, only the main effect of alignment was significant, *F*(1,18) = 22.38, *MSE* = 10.67, *p* < 0.001. The congruency effect in the aligned condition (*M* = 0.89) was greater than that in the misaligned condition (*M* = 0.14). Neither the main effect of part nor its interaction with alignment was significant, *F*s < 1. These latter results again indicate that, compared to Experiment 1, we were better able to control perceptual discriminability between top and bottom parts when we enlarged the spatial separation between them in the misaligned condition.

## General Discussion

The main purpose of the present study was to examine whether differential holistic processing between the top and bottom face parts, measured by congruency effect with the complete design, would be eliminated when parts were equated in terms of perceptual discriminability. In Young et al. (1987), reaction times were longer in the misaligned than aligned condition, and there was an interaction between part and alignment. Rossion (2013) recently suggested that this finding is indicative of a top–bottom asymmetry in the composite effect, where holistic processing is larger for the top than bottom part.

However, differential holistic processing obtained by Young et al. (1987) may have been due to a confound from stimulus discriminability. To avoid this confound, it is necessary to control discriminability between top and bottom face parts. In Experiment 1, our results revealed that participants performed equally well when top or bottom halves were presented in isolation, indicating that top and bottom face halves were equally discriminable perceptually. Furthermore, the results of Experiments 1 and 2 suggest that holistic processing is distributed homogenously within an upright face, consistent with predictions derived from both the template and perceptual field hypotheses, which suggest that upright faces induce a relatively large perceptual spatial window that encompasses the entire face. Our findings are also consistent with predictions based on the attention strategy hypothesis where attentional weights are equal for all face parts.

Given our findings, we suggest that the results from Young et al. (1987), which have been taken as an indication of top-bottom asymmetry, might have been caused by differences in stimulus discriminability. In addition to the physical factor of discriminability, it is worth noting that Rossion (2013) proposed several other factors that may affect homogeneity of holistic processing within a face. For example, there are more fixations at the eye region than at the mouth region (Bombari et al., 2009; Xu and Tanaka, 2013). Moreover, the eye region seems to be more attractive and contains more social information (Baron-Cohen et al., 1997). In contrast, some patients (e.g., prosopagnosia) show less attention to the top half of faces (Orban de Xivry et al., 2008; Ramon et al., 2010).

## Conclusion

The present study was designed to whether there is differential holistic processing within a face. Our findings demonstrate a top/bottom symmetry, not asymmetry, in holistic processing, lending credence to the proposal that representations underlying holistic processing are unitary and homogenous, with equal weighting between top and bottom face parts. Although our results support the general notion of symmetric holistic processing within an upright face, this does not necessarily mean that the magnitude of holistic processing for top and bottom parts cannot be altered. Quite the contrary—recent studies have shown that attention and experience can modulate holistic processing (Chua et al., 2014; Richler and Gauthier, 2014). As another alternative, researchers could also consider the possibility that both the holistic encoding (template) and attention strategy hypothesis are both in operation, such that while representations of upright faces are holistic, its processing can be subject to attentional modulation. For example, in Experiment 2, we enlarged the separation between top and bottom parts in the *misaligned* condition to the point they were separated completely by 4^0^ without any visible overlap (see the two panels on the left in **Figure 6**, p. 17). We speculated that with the complete separation of top and bottom parts in the misaligned condition, participants of Experiment 2 probably had more opportunity to learn, perhaps by constricting more effectively their perceptual field to the top part when it was the target, and thereby minimized the potential interference from the irrelevant, bottom part, especially when the bottom part would elicit an incongruent response. This may be the reason why no significant difference between top and bottom parts was found when we used congruency effect as the dependent measure in Experiment 2. In future studies, we seek to unravel the factors that may modulate holistic processing, especially with respect to predictions based on the holistic encoding versus attention strategy hypotheses.

## Author Contributions

GS contributed to the rationale of the whole experiments, analyzed the data and drafted the manuscript. C-CW found the conflict of the rationale, designed the experiments, collected and analyzed the data. The authors both revised the manuscript and replied to the reviewers.

## Conflict of Interest Statement

The authors declare that the research was conducted in the absence of any commercial or financial relationships that could be construed as a potential conflict of interest. The reviewer EB and handling Editor declared their shared affiliation, and the handling Editor states that the process nevertheless met the standards of a fair and objective review.
